# Identification and Functional Testing of *ERCC2* Mutations in a Multi-national Cohort of Patients with Familial Breast- and Ovarian Cancer

**DOI:** 10.1371/journal.pgen.1006248

**Published:** 2016-08-09

**Authors:** Andreas Rump, Anna Benet-Pages, Steffen Schubert, Jan Dominik Kuhlmann, Ramūnas Janavičius, Eva Macháčková, Lenka Foretová, Zdenek Kleibl, Filip Lhota, Petra Zemankova, Elitza Betcheva-Krajcir, Luisa Mackenroth, Karl Hackmann, Janin Lehmann, Anke Nissen, Nataliya DiDonato, Romy Opitz, Holger Thiele, Karin Kast, Pauline Wimberger, Elke Holinski-Feder, Steffen Emmert, Evelin Schröck, Barbara Klink

**Affiliations:** 1 Institute for Clinical Genetics, Faculty of Medicine Carl Gustav Carus, Technische Universität Dresden, Dresden, Germany; 2 German Cancer Consortium (DKTK), Dresden, Germany; 3 German Cancer Research Center (DKFZ), Heidelberg, Germany; 4 MGZ—Medical Genetics Center, Munich, Germany; 5 Clinic for Dermatology Venerology and Allergology, Göttingen, Germany; 6 National Center for Tumor Diseases (NCT), Partner Site Dresden, Germany; 7 Department of Gynecology and Obstetrics, Medical Faculty and University Hospital Carl Gustav Carus, Technische Universität Dresden, Germany; 8 Vilnius University Hospital Santariskiu Clinics, Hematology, Oncology and Transfusion Medicine Center, Vilnius, Lithuania; 9 State Research Institute Innovative Medicine Center, Vilnius, Lithuania; 10 Masaryk Memorial Cancer Institute, Brno, Czech Republic; 11 Institute of Biochemistry and Experimental Oncology, First Faculty of Medicine, Charles University in Prague, Prague, Czech Republic; 12 Cologne Center for Genomics, Cologne, Germany; 13 Clinic of Dermatology, Rostock, Germany; Cleveland Clinic Genomic Medicine Institute, UNITED STATES

## Abstract

The increasing application of gene panels for familial cancer susceptibility disorders will probably lead to an increased proposal of susceptibility gene candidates. Using *ERCC2* DNA repair gene as an example, we show that proof of a possible role in cancer susceptibility requires a detailed dissection and characterization of the underlying mutations for genes with diverse cellular functions (in this case mainly DNA repair and basic cellular transcription). In case of *ERCC2*, panel sequencing of 1345 index cases from 587 German, 405 Lithuanian and 353 Czech families with breast and ovarian cancer (BC/OC) predisposition revealed 25 mutations (3 frameshift, 2 splice-affecting, 20 missense), all absent or very rare in the ExAC database. While 16 mutations were unique, 9 mutations showed up repeatedly with population-specific appearance. Ten out of eleven mutations that were tested exemplarily in cell-based functional assays exert diminished excision repair efficiency and/or decreased transcriptional activation capability. In order to provide evidence for BC/OC predisposition, we performed familial segregation analyses and screened ethnically matching controls. However, unlike the recently published *RECQL* example, none of our recurrent *ERCC2* mutations showed convincing co-segregation with BC/OC or significant overrepresentation in the BC/OC cohort. Interestingly, we detected that some deleterious founder mutations had an unexpectedly high frequency of > 1% in the corresponding populations, suggesting that either homozygous carriers are not clinically recognized or homozygosity for these mutations is embryonically lethal. In conclusion, we provide a useful resource on the mutational landscape of ERCC2 mutations in hereditary BC/OC patients and, as our key finding, we demonstrate the complexity of correct interpretation for the discovery of “bonafide” breast cancer susceptibility genes.

## Author Summary

Approximately 5–10% of breast/ovarian cancer (BC/OC) cases have inherited an increased risk of developing this malignancy. However, mutations in the two major breast cancer susceptibility genes BRCA1 and BRCA2 explain only 15–20% of all familial BC/OC cases. With the emergence of the high throughput NGS-technology, the number of proposed novel candidate genes for breast cancer predisposition continuously increases. However, a “bonafide” proof of cancer susceptibility requires a detailed characterization of candidate mutations, which we addressed in the current study. Using the DNA repair gene *ERCC2* as an example, we performed a comprehensive multi-center approach, analyzing *ERCC2* mutations in 1000+ patients with hereditary BC/OC. We identified 25 potential candidate mutations for cancer breast cancer susceptibility, some of them affecting *ERCC2* functional activity in appropriate cell-culture based assays. However, a more dissected analysis showed no convincing co-segregation with BC/OC and there was no longer a significant overrepresentation in BC/OC when compared to regionally matched controls instead of the global ExAc variant data base, pointing to the relevance of founder-mutations. In conclusion, we provide a useful resource on the mutational landscape of *ERCC2* mutations in hereditary BC/OC patients and, as our key finding, we highlight the complexity of correct interpretation for the discovery of “bonafide” breast cancer susceptibility genes.

## Introduction

Since it became evident that only 15%-20% of the familial risk for BC/OC can be explained by mutations in the major breast cancer-susceptibility genes *BRCA1* and *BRCA2* [1], the search for additional BC/OC susceptibility loci has been pursued. In times of limited sequencing power this pursuit was based on carefully selected candidate genes which typically came from (i) cancer-associated syndromes (ii) linkage screens in large *BRCA1/2*-negative families and (iii) case–control association studies using single-nucleotide polymorphisms [2,3]. Since sequencing power is no longer an issue, the candidate approach is on its decline and about to be replaced by next generation sequencing (NGS) of large gene panels which, taken together, cover a total of more than 100 genes, only 21 of which have been associated with breast cancer so far [4]. This offers amazing opportunities for detection of novel susceptibility loci but also bears the danger of substantial misuse [4], because variants picked up by these panels are not clinically validated. Therefore, post-marketing data validation is absolutely essential [5]. Rare variants, however, need huge case-control datasets in order to reach the requested statistical significance of P<0.0001 [4]. Until such large datasets become available, variant validation needs to focus on mutations that are clearly deleterious on functional level but still frequent enough to be validated by a few thousand controls. Such recurrent yet harmful variants are best identified by screening various populations for founder mutations. In *NBN*, for example, a protein-truncating variant (c.657del5) has been identified in Eastern Europe, which is sufficiently common to allow its evaluation in a BC/OC case–control study [6]. Also the successful validation of deleterious Polish and Canadian founder mutations in *RECQL* [7] underlines the huge potential of multi-national BC/OC cohorts.

In this study we sequenced 1345 BC/OC cases from 3 different Central- and East European countries with multi-gene panels and identified recurrent founder mutations in *ERCC2*, which were functionally validated in cell-culture based assays. As essential component of transcription factor IIH, the ERCC2 protein is involved in basal cellular transcription [8] and nucleotide excision repair (NER) of DNA lesions [9]. The most known inherited disease associated with bi-allelic mutations in *ERCC2* is Xeroderma pigmentosum type D (XPD, OMIM 278730), a hereditary cancer-prone syndrome characterized by extreme skin photosensitivity and early development of multiple skin tumors [10]. Therefore, *ERCC2* is a plausible candidate gene for cancer susceptibility. On the other hand, bi-allelic mutations in *ERCC2* can also lead to syndromes without increased propensity to tumor development, namely Trichothiodystrophy 1 (TTD; OMIM 601675) and cerebrooculofacioskeletal syndrome (COFS2; OMIM 610756). This indicates that not all functionally relevant *ERCC2* mutations increase cancer susceptibility in their carriers.

## Results and Discussion

### Panel sequencing identifies a broad spectrum of rare variants as well as recurrent founder mutations in *ERCC2*

**Fig 1:**
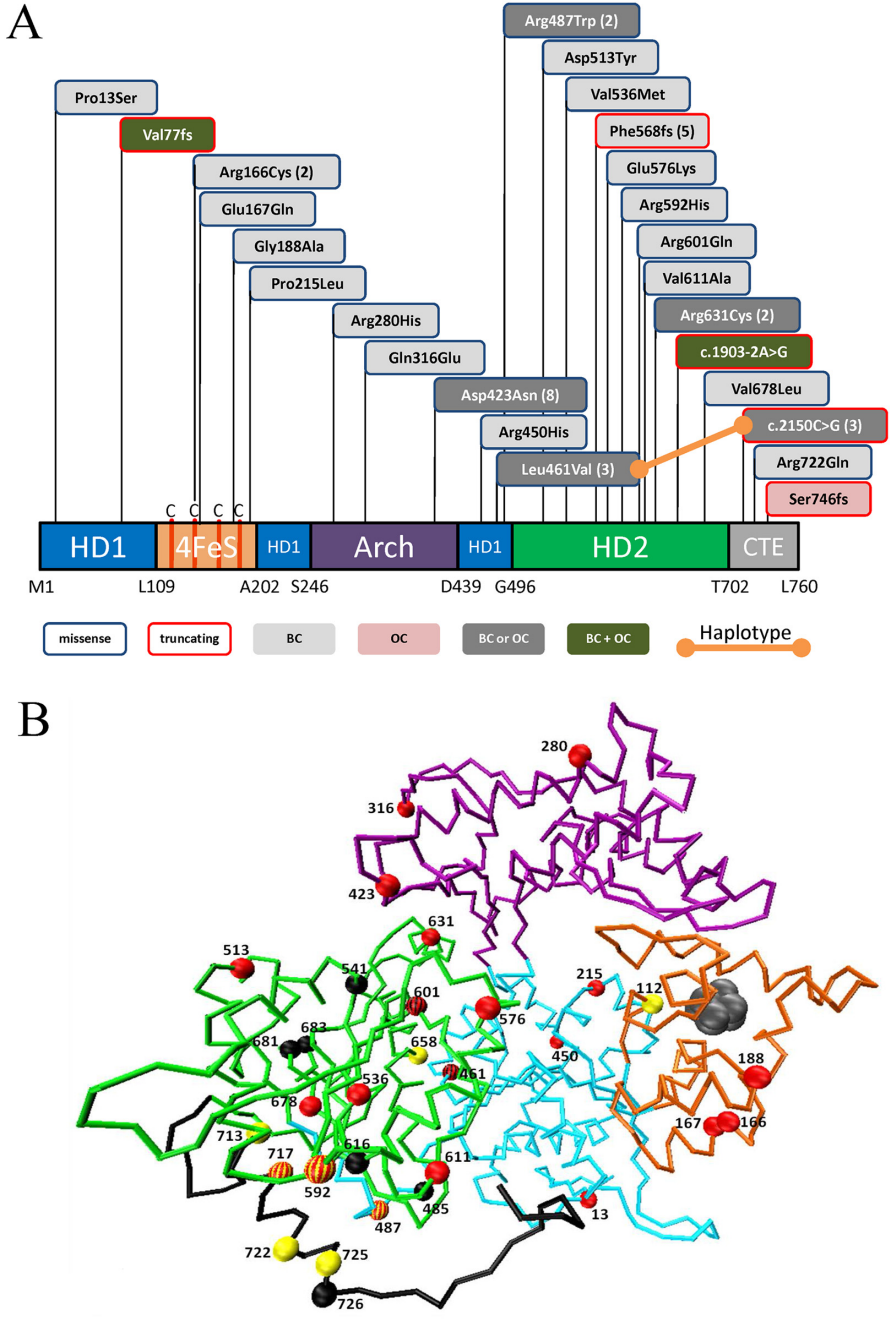
Domain structure and modeling of the ERCC2 mutations. (A) Mutations in the XPD/ERCC2 protein domains. The diagram shows the ERCC2 protein with the four XPD domains shown as HD1 (blue), HD2 (green), FeS (Orange) and Arch (purple). The human enzyme has a C-terminal (grey) extension (CTE) that probably forms an interaction surface with the p44 protein. Disease-relevant *ERCC2* mutation sites are indicated in boxes (blue or red frame: missense or truncating mutation, respectively; fillings: light-gray, cases with breast cancer (BC); pink, case with ovarian cancer only (OC); dark-gray: cases with either breast- or ovarian cancer (BC or OC); dark-green, patients with both breast- and ovarian cancer (BC + OC)). Numbers in brackets indicate recurrent mutations. (B) Structural placement of mutations on a C-alpha trace model of human ERCC2. The residues targeted by HBOC-causing mutations are represented as space-filled red spheres. Xeroderma pigmentosum (XP) and trichothiodystrophy (TTD) disease causing mutations sites as reported in ClinVar are shown in yellow and black spheres. Missense variants at residue position 423, 461, 487, 568, 461 and 722 have been found in both BC/OC as well as XP (red-yellow spheres) and TTD (red-black spheres) patients.

Within the entire set of 1345 BC/OC index cases, we have detected three different frame-shift (fs) mutations [p.(Val77fs), p.(Phe568fs) and p.(Ser746fs)], one splice-acceptor site mutation (c.1903-2A>G), one nucleotide exchange that activates a cryptic splice site (c.2150C>G) and 20 rare missense mutations (Table 1, Fig 1). Whereas 14 mutations were unique (2 fs, 1 splice-site, 11 missense), 11 mutations (1 fs, 1 splice-affecting, 9 missense) have been found in 43 independent families. The most frequent mutation was p.(Asp423Asn) identified in 8 carriers from Lithuania and one from the Czech Republic. The common polymorphisms p.(Lys751Gln) and p.(Asp312Asn) have each been encountered in approximately 64% of our cases; since these variants have been considered to be functionally irrelevant [11], we did not include them in our functional study. Among the 20 rare missense variants reported in Table 1, thirteen are predicted by various computer algorithms to be pathogenic (Table 1 and S4 Table). Further computational analysis of the conservation (PhyloP) and depletion (CADD) scores [12] for the mutated nucleotides strongly supported pathogenicity for these variants (S2 Fig). Mapping the mutated AA positions onto the ERCC2 protein structure revealed a widespread distribution pattern (Fig 1). Residues 13, 450, 461, 513, 536, 576, 592, 601, 611, 631, 678 cluster at the helicase motifs of the HD1 and HD2 catalytic domains and residues 166, 167, 188, 215, 280, 316, 423, 487, 722 locate at the TFIIH transcription factor complex binding domains (Arch, FES, and C-terminal). XPD-causing mutations located at the HD2 domain have been shown to inactivate helicase repair capability without disrupting protein structure. Mutations causing trichothiodystrophy (TTD, OMIM 601675), on the other hand, are located well away from the catalytic site of the enzyme and destabilize ERCC2 structure and TFIIH protein interactions [13–15]. We suggest that BC/OC relevant mutations might affect both—catalytic activity as well as protein stability.

**Table 1:** Mutations and rare variants in ERCC2 identified through panel sequencing of individuals with familial breast and/or ovarian cancer. AA = amino acid; N = sample size; n.a. = not applicable; n.t. = not tested; CZ = Czech Republic, GE = Germany, LT = Lithuania. The cumulative assessment is based on the results of various effect prediction algorithms; details see S4 Table.

Variant description					Predicted effect	Functional effect		BC/OC cases				
Position	Exon	Nucleotide change	AA change	rs-ID	Cumulative assessment	Complementation of NER-deficient cells	Negative modulation of transcription	GE	CZ	LT	total	Tumor type
								N = 587	N = 353	N = 405	N = 1345	
hg19	(23)	NM_000400.3	max = 760 aa									
19:45873459	2	c.37C>T	p.(Pro13Ser)	-	pathogenic	n.t.	n.t.	1	0	0	1	BC
19:45872203	4	c.230_231delTG	p.(Val77Alafs)	-	n.a.	n.t.	n.t.	0	1	0	1	BC+OC
19:45868194	7	c.496C>T	p.(Arg166Cys)	-	pathogenic	n.t.	n.t.	0	0	2	2	BC
19:45868191	7	c.499G>C	p.(Glu167Gln)	rs367829012	benign	n.t.	n.t.	1	0	0	1	BC
19:45868127	7	c.563G>C	p.(Gly188Ala)	-	benign	n.t.	n.t.	1	0	0	1	BC
19:45867756	8	c.644C>T	p.(Pro215Leu)	-	pathogenic	n.t.	n.t.	0	0	1	1	BC
19:45867354	10	c.839G>A	p.(Arg280His)	-	pathogenic	n.t.	n.t.	0	0	1	1	BC
19:45867247	10	c.946C>G	p.(Gln316Glu)	-	benign	n.t.	n.t.	1	0	0	1	BC
19:45860928	13	c.1267G>A	p.(Asp423Asn)	rs143710107	benign	no	yes	0	1	8	9	4xBC, 5xOC
19:45860760	14	c.1349G>A	p.(Arg450His)	rs146632315	pathogenic	yes	no	2	0	0	2	BC
19:45860626	15	c.1381C>G	p.(Leu461Val)	rs121913016	benign	yes	yes	3	0	0	3	2xBC, 1xOC
19:45860548	15	c.1459C>T	p.(Arg487Trp)	rs562132292	pathogenic	no	yes	0	0	4	4	2xBC, 2xOC
19:45858929	16	c.1537G>T	p.(Asp513Tyr)	-	pathogenic	yes	yes	1	0	0	1	BC
19:45858047	17	c.1606G>A	p.(Val536Met)	rs142568756	pathogenic	yes	yes	2	0	0	2	BC
19:45856554	18	c.1703_1704delTT	p.(Phe568fs)	-	pathogenic	no	no	1	3	1	5	BC
19:45856532	18	c.1726G>A	p.(Glu576Lys)	rs201165309	pathogenic	n.t.	n.t.	1	0	0	1	BC
19:45856397	19	c.1775G>A	p.(Arg592His)	rs147224585	pathogenic	yes	no	1	0	7	8	BC
19:45856370	19	c.1802G>A	p.(Arg601Gln)	rs140522180	pathogenic	yes	yes	2	1	0	3	BC
19:45856074	20	c.1832T>C	p.(Val611Ala)	-	benign	n.t.	n.t.	0	1	0	1	BC
19:45856015	20	c.1891C>T	p.(Arg631Cys)	rs144511865	pathogenic	no	no	1	0	1	2	1xBC, 1xOC
19:45855909	IVS 20	c.1903-2A>G	splice site	-	n.a.	n.t.	n.t.	1	0	0	1	BC+OC
19:45855778	21	c.2032G>C	p.(Val678Leu)	-	benign	n.t.	n.t.	0	0	1	1	BC
19:45855507	22	c.2150C>G	splice effect	rs144564120	pathogenic	n.t.	n.t.	3	0	0	3	2xBC, 1xOC
19:45855492	22	c.2165G>A	p.(Arg722Gln)	rs138569838	pathogenic	n.t.	n.t.	0	1	0	1	BC
19:45854932	23	c.2238delA	p.(Ser746fs)	-	n.a.	yes	yes	1	0	0	1	OC

### Functional testing identifies *ERCC2* mutations with deleterious effects on protein level

**Fig 2:**
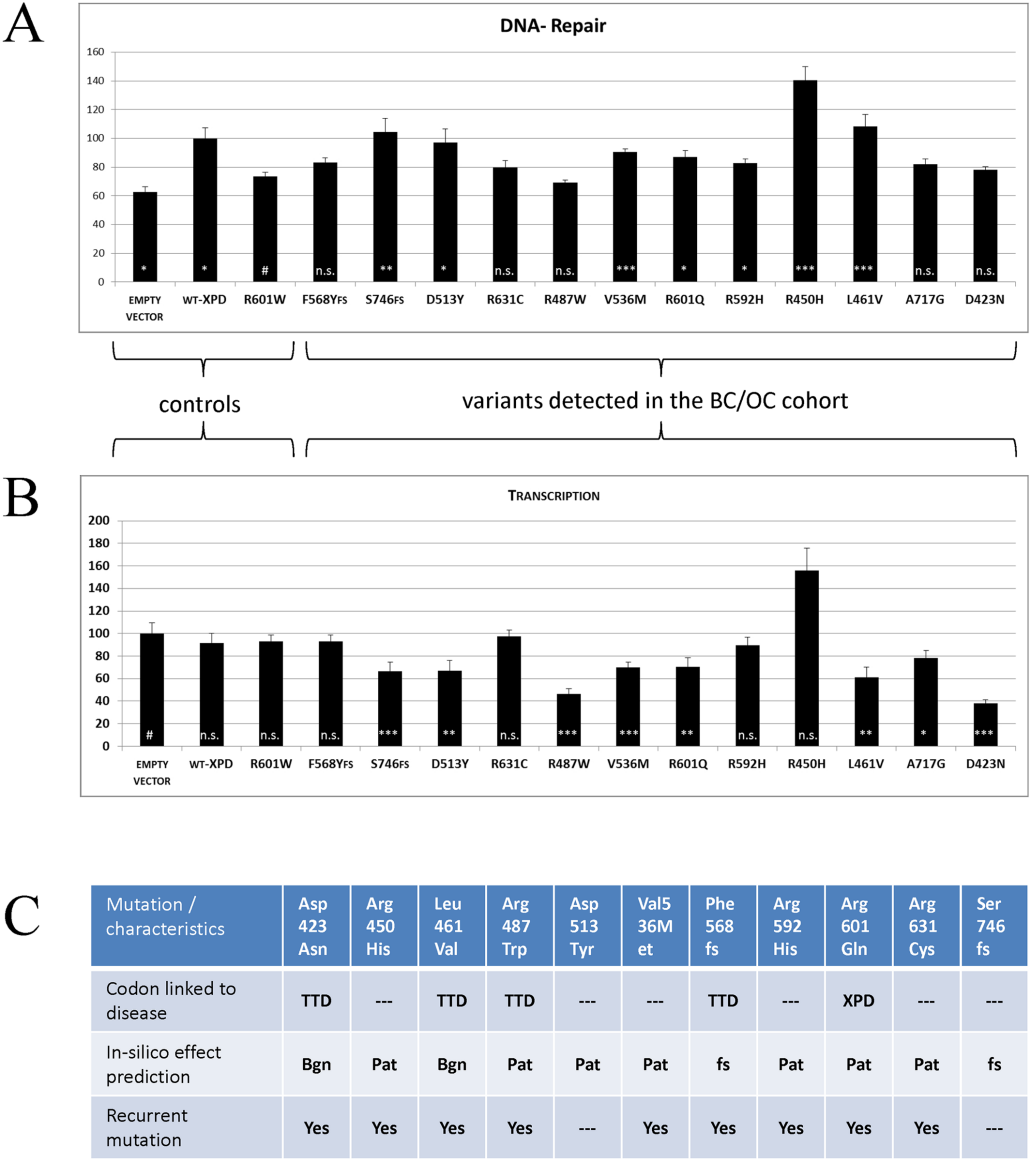
Nucleotide excision repair (NER) capacity and Transcriptional activity of breast cancer associated XPD/ERCC2 variants. (A) Several XPD/ERCC2 variants cloned into an expression vector were analyzed regarding to complementation of *ERCC2*-defective XP6BE cells overexpressing the NER-deficient R601W XPD mutant [15] (normalization for overexpression artifacts). Black bars indicate the mean relative repair capacity (in %, WT-XPD was set to 100%) of an UV irradiated firefly luciferase reporter gene plasmid (UVC 1000 J/m^2^) obtained by host cell reactivation (n>6 in triplicates). Red lines mark the range between DNA-repair levels of empty vector, i.e. residual repair activity of the cells, and WT-XPD, i.e. 100% repair capacity. (B) Dominant modulation of firefly luciferase reporter gene expression (without irradiation) via overexpression of XPD/ERCC2 BC/OC-associated variants was estimated in the transcriptionally-proficient but repair-deficient XPD/ERCC2-defective XP6BE cells. Black bars indicate the mean relative reporter gene expression (in %, empty vector control was set to 100%), obtained by CMV-promotor driven basal transcription (n>6 in triplicates). Error bars indicate the standard error of the mean. Significance levels were calculated, after pairwise testing for normal distribution of the values, using appropriate statistical tests for comparison of two groups (T-Test or U-Test, # = reference group, *** = p<0.001, ** = p<0.01, * = p<0.05, n.s. = not significant). (C) Additional characteristics of the mutations tested for repair efficiency and transcriptional activity.

So far, 11 variants (9 recurrent founder mutations and 2 unique variants; Fig 2C) were tested in functional assays for nucleotide excision repair (NER) capability (Fig 2A) as well as transcription (Fig 2B). Whereas six out of the 11 BC/OC-associated *ERCC2* variants tested in this study, have not yet been linked to any disease [AA positions 423, 450, 513, 536, 631, 746], five AA positions have already been found to be mutated in either TTD [AA 461 [16], 487 [17], 568 [18,19], 592 [20]] or XPD [AA 601 [21]] (Figs 1B and 2C). According to our functional assays, four ERCC2 protein variants [p.(Asp423Asn), p.(Arg487Trp), p.(Phe568Tyrfs) and p.(Arg631Cys)] failed to enhance functional NER of an UV-treated reporter gene plasmid indicating the impairment of ERCC2 repair capacity. The remaining seven tested variants retained some NER capability (Fig 2A). Concerning transcription, we detected a dominant negative influence of seven ERCC2 protein variants [p.(Asp423Asn), p.(Leu461Val), p.(Arg487Trp), p.(Asp513Tyr), p.(Val536Met), p.(Arg601Gln), p.(Ser746fs)] on reporter gene expression (Fig 2B) indicating transcription blocking. In summary, 10 of 11 mutations display diminished excision repair efficiency and/or decreased transcriptional activation capability, with p.(Asp423Asn) and p.(Arg487Trp) being the variants with the highest impact on protein function.

### The majority of the *ERCC2* mutations are founder mutations

The hallmarks of a founder mutation are recurrent appearance, population specificity and haplotype sharing. As to recurrent appearance, 11 out of 25 *ERCC2* mutations were seen at least twice in our BC/OC cohort (last column in Table 1). Among the 11 recurrent variants, 5 were identified exclusively in one of the three populations tested in this study (e.g. p.(Arg487Trp): 4x LT only) and another 5 were significantly overrepresented in one of the 3 populations (e.g. p.(Asp423Asn): 8x LT, 1x CZ, 0x GE). For two of the population-enriched recurrent founder mutations, we could also demonstrate haplotype sharing: (i) the mutation c.1381C>G (rs121913016) always co-occurred and co-segregated with mutation c.2150C>G (rs144564120), a haplotype which has been observed repeatedly in TTD/XPD patients [9,16,22]. (ii) In almost all cases (10/11) the frame-shift mutation c.1703_1704delTT co-occurred with the c.1758+32C>G polymorphism (rs238417). Furthermore, these two variants are only 84 nt apart from each other and all NGS-reads covering both variants showed these variants simultaneously, i.e. these variants are definitely localized in *cis* on the same DNA molecule.

### Even small region-specific control cohorts outnumber huge public variant databases

**Fig 3:**
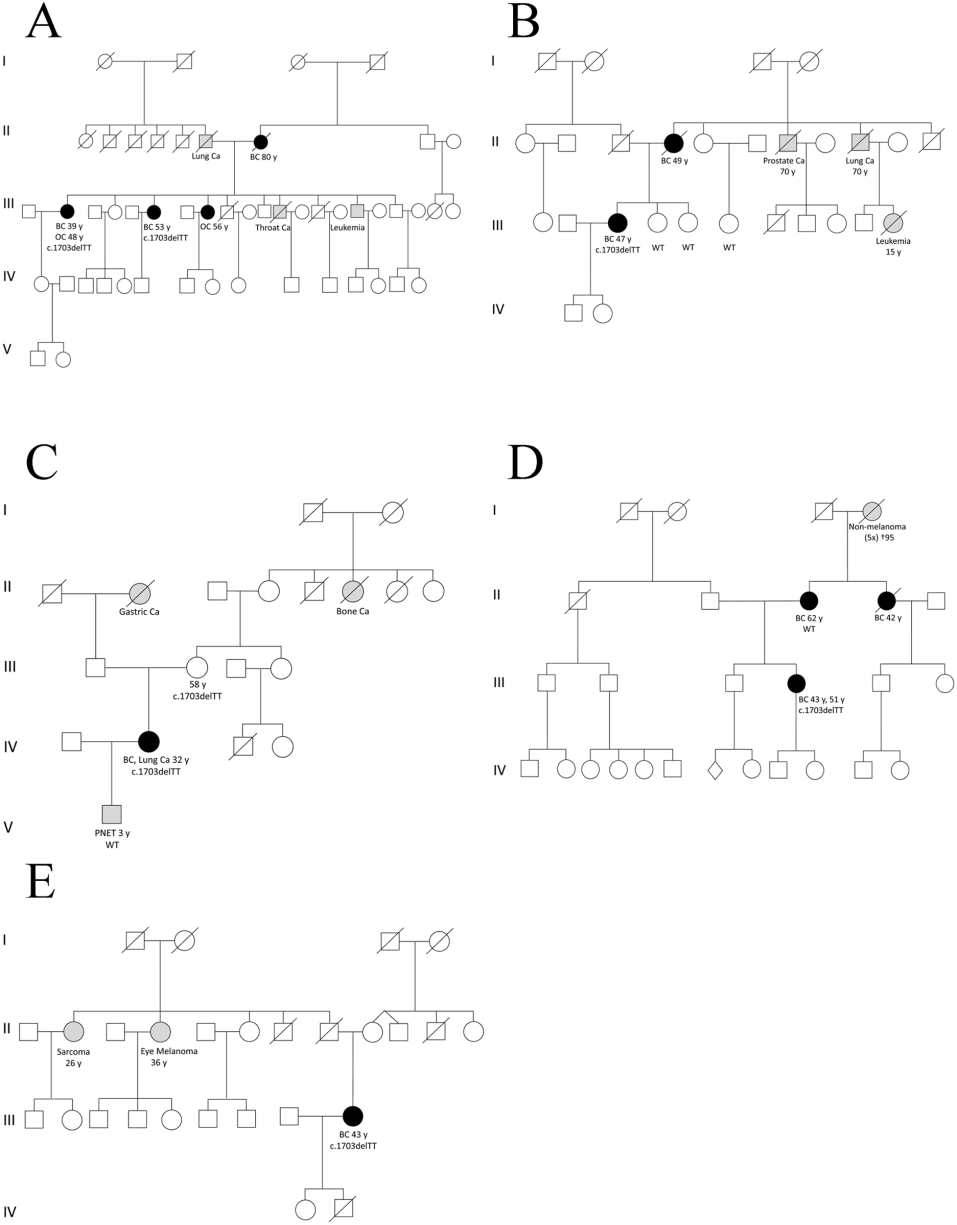
*ERCC2* frameshift mutation c.1703_1704delTT (p.Phe568fs) in familial breast and ovarian cancer pedigrees. Individuals with breast cancer (BC), ovarian cancer (OC) or both (BC, OC) are shown as circles filled in black. Individuals tested positive for the familial mutation are indicated in detail; those with WT (wild-type) have been tested negative. All affected individuals with BC or OC not tested for germline mutations in ERCC2 were either deceased or refused testing. (A) German, (B) Lithuanian and (C-E) Czech pedigrees.

In the variant discovery phase of this project, the frequencies of *ERCC2* mutations found in the BC/OC cohort were compared to the corresponding frequencies in public databases provided by the NHLBI Exome Sequencing Project (ESP) and the Exome Aggregation Consortium (ExAC). As shown in Table 2, some intriguing mutations, like p.(Phe568fs) and p.(Asp423Asn), have very low frequencies according to ExAC, suggesting significant odds ratios (OR). As a first proof of principle measure, we performed segregation analysis. However, none of our recurrent *ERCC2* mutations showed convincing co-segregation with BC/OC (Fig 3). Moreover, as soon as a small number of population-specific control probands has been sequenced, it became clear that almost all founder mutations in the BC/OC cohort showed similar frequencies in the ethnically matching control cohorts. The only exception so far is the Lithuanian mutation p.(Arg487Trp), which was found 4 times in the Lithuanian BC/OC cohort and not (yet) in the corresponding control cohort (Table 2). With just above 100 individuals this cohort is way too small to be of any statistical relevance. Therefore, the acquisition of additional samples is mandatory. But even in this very early phase of variant (de-)validation it becomes evident that regionally matching control cohorts–as small as they may be–are superior to any huge global cohort. Since genotypic data allow to locate the geographic origin of a given individual within a few hundred kilometers [23], the term “regionally matching” should be defined as “less than ca. 300 km distance from the recruitment center”. As a consequence, regionally matching controls are even superior to population-specific controls, because populations do mix, especially in regions close to national borders. The p.Phe568fs mutation, for example, has been seen only once in a German BC/OC index case and never in the 1844 German controls. Based on population-specific data we would have been very excited about this finding. But the German case was recruited in Dresden, close to the Czech border, and in Prague, 118 km away, the same mutation has been found twice in a small control cohort of only 105 non-cancer females. This underlines the importance of regional controls and multi-national studies for reliable variant validation.

**Table 2:** *ERCC2* allele frequencies (%) in BC/OC patients and corresponding control cohorts. The allele frequency is counted on the basis of sample size (in brackets) and number of observed cases (see Table 1) with hetero- and homozygosity.

AA / nt change	CZ	CZ	LT	LT	GE	GE	ExAc
(N = 25)	BC/OC	Ctrl	BC/OC	Ctrl	BC/OC	Ctrl	vers. 0.2
	[353]^a^	[453]^b^	[405]	[103]	[587]^c^	[1844]^d^	[variable]^e^
Pro13Ser	0	0	0	0	0.0851	0	0
Val77Alafs	0.1416	0	0	0	0	0	0
Arg166Cys	0	0	0.2469	0	0	0	0
Glu167Gln	0	0	0	0	0.0851	0	0.0033
Gly188Ala	0	0	0	0	0.0851	0	0
Pro215Leu	0	0	0.1234	0	0	0	0
Arg280His	0	0	0.1234	0	0	0	0.0072
Gln316Glu	0	0	0	0	0.0851	0	0.0152
Asp423Asn	0.1416	0.1104	0.9876	1.456	0	0.0542	0.0248
Arg450His	0	0	0	0	0.1704	0.0813	0.0214
Leu461Val	0	0	0	0	0.2553	0.1356	0.1345
Arg487Trp	0	0	0.4938	0	0	0	0.0034
Asp513Tyr	0	0	0	0	0.0851	0	0
Val536Met	0	0	0	0	0.1704	0	0.0231
p.Phe568fs	0.4249	0.4415	0.1234	0	0.0851	0	0.0093
Glu576Lys	0	0	0	0	0.0851	0.0542	0.0008
Arg592His	0	0	0.8642	0	0.0851	0	0.0332
Arg601Gln	0	0.1104	0	0	0.1704	0.0542	0.0175
Val611Ala	0.1416	0	0	0	0	0	0.0042
Arg631Cys	0	0	0.1234	0	0.0851	0	0.0025
c.1903-2A>G	0	0	0	0	0.0851	0	0
Val678Leu	0	0	0.1234	0	0	0	0
c.2150C>G	0	0	0	0	0.2553	0.0813	0.0349
Arg722Gln	0.1416	0	0	0	0	0	0.0067
p.Ser746fs	0	0	0	0	0.0851	0	0

BC/OC = index cases with breast- and/or Ovarian cancer; Crtl = healthy or non-cancer related individuals; CZ = Czech Republic, GE = Germany, LT = Lithuania; AA = Amino acid; nt = nucleotide; ExAC = Exome Aggregation Consortium, Cambridge, MA (URL: http://exac.broadinstitute.org) [accessed May 2015];

^a^ 28 samples from Brno (TruSight-Cancer) + 325 samples from Prague [24,25] (custom panel with 581 genes);

^b^ 105 female non-cancer samples from Prague [25,26] (custom panel with 581 genes) + 108 female non-cancer samples from Brno, sequenced in pools with the TruSight-Cancer panel + 240 non-cancer samples from Prague, sequenced in pools with the TruSight-Cancer panel;

^c^ 271 samples from Dresden + 316 samples from Munich (MGZ), all sequenced with the TruSight-Cancer panel;

^d^ 1629 individual exome samples from the Cologne Center for Genomics (CCG) + 79 individual non-BC/OC TruSight-One samples from Dresden + 136 individual non-BC/OC TruSight-Cancer samples from Dresden and Munich (MGZ);

^e^ Since the exome data have been collected from various sources with various enrichment strategies, the sample size varies for each variant. Each allele frequency has been calculated with the corresponding sample size for that allele.

### *ERCC2* mutations with tumorigenic relevance are probably located in very small and scattered areas of the protein

Due to its involvement in DNA repair and due to encoding a helicase like *RECQL* [7], *ERCC2* is a plausible gene candidate for familial cancer susceptibility. Bi-allelic mutations in *ERCC2*, however, can cause the cancer-prone disease XPD as well as the “non-cancer”-disease TTD [27] and there is no evident genotype-phenotype correlation [19]. The pathogenic p.(Arg112His) mutation, for example, has been identified in TTD patients as well as in a patient with major features of XPD [19]. Furthermore, impairment of DNA repair capacity is not correlated with tumor burden: the mutation p.(Phe568Tyrfs), for example, has been identified in non-cancer TTD patients twice, but not once in cancer-prone XPD patients, although this study (Fig 2) as well as a previous study [19] clearly show diminished repair capability of this frameshift variant. From these observations we have to conclude that a limited subset of mutations in *ERCC2* might predispose to cancer but these mutations are not likely to cluster in a defined area of the gene nor do they necessarily affect a specific sub-function of the ERCC2 protein. Therefore, cancer predisposing *ERCC2* mutations are very likely to be discovered only on the basis of familial co-segregation with cancer and overrepresentation in cancer cohorts vs. region-specific controls.

### The incidence of *ERCC2*-related diseases is not in line with the frequency of deleterious founder mutations in the corresponding populations

Although the founder mutations tested in this study may not predispose to BC/OC they still confer carrier status for the recessive disorders XPD (OMIM 278730), TTD (OMIM 601675) and COFS2 (OMIM 610756). Even the TTD-causing mutation p.(Phe568fs) alone has been detected in 7 of 806 samples from the Czech Republic (CZ), i.e. the frequency of heterozygous carriers of this mutation is approx. 0.86%. According to Hardy-Weinberg equilibrium model, this would result in a TTD incidence of 1/30.000. Based on combined data from the DNA repair diagnostic centers in France, West-Germany, Italy, the Netherlands and the United Kingdom the actual incidence for TTD is 1.2 per million [28]. Since it is reasonable to assume that (i) a TTD incidence of 1/30.000 would not be missed by the clinical geneticists in CZ and (ii) the publications reporting p.(Phe568fs) as TTD-causing [9,19] are not wrong, there is one logical explanation for the discrepancy between allele frequency and disease incidence: homozygosity for p.Phe568fs is embryonic lethal. This is in-line with the observation that complete loss of ERCC2 activity is not compatible with life in homozygous knock-out mice [29] and it is also consistent with the observation that all XPD and TTD patients tested so far have residual ERCC2 activity [30]. Since an elevated TTD/XPD incidence has not been reported in Lithuania either, we can assume that homozygosity of the frequent Lithuanian founder mutation p.(Asp423Asn) (Table 2), which clearly displayed functional deficiency in our experiments (Fig 2), is embryonic lethal as well.

In conclusion, this multi-national study of *ERCC2* mutations in patients with familial BC/OC and regionally matching controls identified and functionally verified a broad spectrum of unique and recurrent *ERCC2* mutations. Although the frequent founder mutations are not very likely to predispose to BC/OC, some mutations, like p.(Val77Alafs), that are unique to the BC/OC cohort are worth to be considered in future large-scale association studies.

## Materials and Methods

### Ethics statement

Informed written consent was obtained from all patients and the study was approved by the Local Research Ethics Committee (EK 162072007).

### Subjects, families and pedigrees

We enrolled affected individuals from 587 German BC and BC/OC pedigrees with hereditary gynecological malignancies through a genetic counseling program at two centers (Dresden, Munich) from the “German Consortium for hereditary breast- and ovarian cancer” (GC-HBOC) and at the Medical Genetics Center (MGZ) in Munich. Additional 131 BC- and 136 BC/OC families were collected at the Vilnius University Hospital Santariskiu Klinikos in Vilnius, Lithuania and 28 BC/OC families were gathered in the Czech Republic at Brno. The Czech Prague subgroup involved 325 BC patients negatively tested for presence of pathogenic *BRCA1* and *BRCA2* variants [24] and 105 non-cancer controls analyzed as described recently [25,26], and additional 240 controls [26] sequenced in pools. The BC pedigrees fulfilled the criterion that at least three affected females with breast cancer but no ovarian cancers were present (breast cancer pedigrees). In the BC/OC pedigrees, at least one case of breast and one ovarian cancer had occurred. All individuals with variant *ERCC2* alleles were checked for mutations in 10 BC/OC core genes defined by GC-HBOC (*ATM*, *BRCA1*, *BRCA2*, *CDH1*, *CHEK2*, *NBN*, *PALB2*, *RAD51C*, *RAD51D* and *TP53*). Informed consent was obtained from all people participating in the study, and the experiments were approved by the ethics committees of the institutions contributing to this project.

### TruSight-Cancer panel sequencing

DNA was obtained from peripheral blood of all patients. For panel enrichment approximately 85 ng genomic DNA was required. We used the TruSight Cancer Illumina kit (Illumina), which targets the coding sequences of 94 genes associated with a predisposition towards cancer (S1 Table), following the manufacturer's instructions. Sequencing was carried out on an Illumina MiSeq instrument as 150 bp paired-end runs with V2 chemistry. Reads were aligned to the human reference genome (GRCh37/hg19) using BWA (v 0.7.8-r455) with standard parameters. Duplicate reads and reads that did not map unambiguously were removed. The percentage of reads overlapping targeted regions and coverage statistics of targeted regions were calculated using Shell scripts. Single-nucleotide variants and small insertions and deletions (INDELs) were called using SAMtools (v1.1). We used the following parameters: a maximum read depth of 10000 (parameter -d), a maximum per sample depth of 10000 for INDEL calling (parameter -L), adjustment of mapping quality (parameter -C) and recalculation of per-Base Alignment Quality (parameter -E). Additionally, we required putative SNVs to fulfill the following criteria: a minimum of 20% of reads showing the variant base and the variant base is indicated by reads coming from different strands. For INDELs we required that at least 15% of reads covering this position indicate the INDEL. Variant annotation was performed with snpEff (v 4.0e) and Alamut-Batch (v 1.3.1) based on the RefSeq database. Only variants (SNVs/small INDELs) in the coding region and the flanking intronic regions (±15 bp) were evaluated.

### Custom breast cancer panel sequencing

The data related to the ERCC2 gene in this study were retrieved from the custom-made gene panel sequencing analysis described recently [25]. Briefly, genomic DNA was obtained from a peripheral blood of 325 BC Czech patients from the Prague area that were negatively tested for a presence of pathogenic variants in the *BRCA1* or *BRCA2* gene previously [24]. The frequency of population-specific variants was assessed by a concurrent analysis of 105 control DNAs obtained from non-cancer individuals [26]. One μg of genomic DNA was used for library construction. The DNA was fragmented by ultrasonication and edited for SOLiD sequencing. Target DNA enrichment was performed by a custom solution-based sequence capture (SeqCap EZ Choice Library, Roche) according to the NimbleGenSeqCap EZ Library SR User's Guide (Version 4.2, Roche). Five hundred and ninety targeted genes include 141 genes that code for known proteins involved in DNA repair and DNA damage response pathways, and an additional set of genes retrieved from Phenopedia at HuGE Navigator16 web site associated with “breast neoplasms” (assessed February 2012). Captured libraries were sequenced on SOLiD4 system. Finally, exonic regions of 581 genes were captured successfully with sufficient coverage. Reads were aligned to the human reference genome (GRCh37/hg19) using Novoalign (CS 1.01.08) with standard parameters. Conversion of SAM to BAM format was performed with SAMtools (0.1.8). Single-nucleotide variants and small insertions and deletions (INDELs) were called using SAMtools (0.1.8). Variant annotation was performed with ANNOVAR [31]. For final evaluations, small INDELs, intronic variants flanking ± 2 bp to exon borders, and rare SNPs (presented in 1000 genome or exome sequencing (ESP) projects with frequency <1%) were considered.

### Sanger sequencing

Validation of *ERCC2* variants in probands and family members was performed by classical Sanger sequencing. Additional DNAs from 8 HBOC patients affected by malignant melanoma (5 cases) or presence of melanoma in other family members (3 cases) were analyzed for the complete *ERCC2* coding region. *ERCC2* exons were amplified with intronic primers (S2 Table) and sequenced using the ABI Prism Terminator Cycle Sequencing Ready Reaction Kit (Applied Biosystems). Genomic DNA (50 ng) containing 1x PCR Master Mix (Qiagen) and 0.25 μM of each forward and reverse primers in 15 μl reaction volume was subjected to PCR amplification for 25 cycles (30 sec at 95°C, 30 sec at 64°C and 30 sec at 72°C).

### Functional validation of *ERCC2* variants

#### Variant cloning

Wild type *ERCC2* cDNA was amplified from reverse transcribed mRNA isolated from fibroblasts derived from healthy donors (RevertAid H Minus First strand cDNA synthesis kit; Thermo scientific, Waltham, MA, USA) using forward (5’TTAGGTACCATGA AGCTCAACGTGGACG) and reverse (5‘ TTATCTAGATCAGAGCTGCTGAGCAATCT) primers and cloned into the pJET1.2/blunt vector (CloneJET PCR Cloning Kit; Life technologies, Waltham, MA, USA). These primers carry *Kpn*I and *Xba*I restriction sites to release *ERCC2* cDNA by double restriction enzyme digestion (Life technologies). The *ERCC2* cDNA was purified from agarose gels using the Wizard SV Gel and PCR Clean-Up System (Promega, Klaus, Austria) and cloned into the pcDNA3.1(+) mammalian expression vector (Life technologies) and subsequently transformed into DH5α *E*.*coli* cells. Colony PCR (using T7 and M13 primers) and Sanger sequencing of the entire gene was performed using the BigDye Terminator v3.1 Cycle Sequencing Kit (Life technologies, for primers see S3 Table).

For generation of the *ERCC2* variants, site directed mutagenesis was applied using Phusion High-Fidelity DNA Polymerase (Life technologies) and specific primer pairs in either the classical protocol (for variants Ser746FS and D513Y, Stratagene) or an optimized site-directed method (all other variants, for primers see S3 Table). For the latter, template (100 ng *ERCC2* in pcDNA3.1(+)) was first subjected to dam methylation using dam methyl transferase (NEB, Frankfurt a. M., Germany). Afterwards, a first PCR was conducted with the forward-primer using Phusion polymerase (Life technologies) in a 2-Step PCR protocol with 5 minutes of annealing and elongation at 72°C for 18 cycles. Then over-night enzyme digestion with *Dpn*I (Life technologies) was followed by ethanol precipitation. A second PCR using reverse primers (same conditions) was performed with this template and ethanol precipitated. The final reaction product was subject to transformation of DH5α *E*.*coli* cells. Positive clones were verified by Sanger sequencing as described above.

#### Assay set-up

The host cell reactivation (HCR) assay measures the amount of nucleotide excision repair (NER) in actively transcribed genes. This dual reporter gene assay deploys the turnover rate of firefly luciferase substrate as readout for the NER capacity of host cells transfected with the (UV-) damaged reporter gene plasmid encoding for firefly luciferase [32]. HCR can be used for DNA repair capacity assessment of NER deficient host cells transfected with DNA repair gene variants as well as for measuring in situ transcription using non-irradiated firefly luciferase reporter gene plasmids [33,34].

*ERCC2*-deficient XP6BE-SV-immortalized fibroblasts were a generous gift of K.H. Kraemer (NIH, Bethesda, MD, USA) and harbor two differently mutated *ERCC2* alleles [p.Arg683Trp and an in-frame deletion of amino acids (AA) 36–61] [9]. XP6BE cells were transfected using Attractene Transfection Reagent (Qiagen, Hilden, Germany) according to the manufacture’s advice, with plasmids coding for firefly luciferase (100 ng), renilla-luciferase (50 ng) and an empty pcDNA3.1(+) vector or XPD-variants cloned into the pcDNA3.1(+) expression vector (100 ng) (for cloning see above). The plasmid coding for firefly luciferase was divided into two fractions prior to transfection. One fraction was irradiated with 1000 J/m2 of UVC light, a second fraction stayed untreated. The non-irradiated renilla-luciferase plasmid serves as an internal control for normalization of transfection efficacy.

After incubation of transfected XP6BE cells for one day (37°C, 5% CO_2_), which allows sufficient repair of the UV-photoproducts and protein expression of the luciferases, cells were lysed and analyzed using Dual-Luciferase Reporter Assay System (Promega, Klaus, Austria). The luminescence measurements were performed in a white Glomax 96 microplate using the Glomax luminometer (Promega, Klaus, Austria).

The relative repair capacity is estimated using this formula:

$$repair\;\left(\%\right)=\frac{\text{mean }\left(\text{irradiated firefly}/\text{renilla per well}\right)}{\text{mean }\left(\text{unirradiated firefly}/\text{renilla per well}\right)}\;x\;100$$

The repair capacity of XP6BE cells transfected with the wild type *ERCC2* cDNA containing expression vector was set to 100%.

Transcriptional activity was calculated as the amount of firefly luciferase expression from non-irradiated plasmids in XP6BE cells transfected either with wild type *ERCC2* or breast cancer associated *ERCC2* variants containing expression vectors relative to the amount of firefly luciferase expression in XP6BE cells transfected with the empty expression vector. The latter was set to 100%. Every experiment (NER capacity as well as transcription) was conducted at least six times in triplicates.

### Modeling of ERCC2 protein structure

#### Structural modeling of the ERCC2 variants

Homology modeling of the human ERCC2 protein was performed with SWISS-MODEL (ExPASy). The crystal structure of the ATP-dependent DNA helicase Ta0057 from *Thermoplasma acidophilum* (RCSB:4A15, UniProt:Q9HM14) was used as template structure for modeling. Predicted models for the residue changes of the detected missense mutations in *ERCC2* were displayed and analyzed using Visual Molecular Dynamics (VMD) (S1 Fig). The predicted models were superimposed onto the Ta0057 structure with the MulitSeq tool integrated in VMD.

#### In-silico interpretation of missense variants

The probability of effect of non-synonymous mutations in *ERCC2* was calculated by the amino acid (AA) substitution prediction methods SIFT, PolyPhen2, Provean, Mutation Taster, MAPP, and AGVD (S4 Table). Based on these data, a summarizing rating was assessed (last column in S4 Table and Table 1). Distribution of PhyloP and Grantham scores [35] for dbSNP, ClinVar and all variants identified in *ERCC2* were analyzed. Statistical probability scores of PhyloP and Grantham scores and analysis of distribution plots are provided (S4 Table and S2 Fig).

### AA conservation alignment

A multiple alignment of ERCC2 AA sequences was done according to HomoloGene (NCBI) in order to assess the AA conservation of the detected variants in 20 species with homologous proteins (S3 Fig).

## Supporting Information

S1 Fig ERCC2 domain structure and overlay of ERCC2 missense mutations Arg478Trp and Asp423Asn. A) Schematic showing the domain structure and canonical motifs of human ERCC2. Helicase motor domains HD1 (blue) and HD2 (green) form the DNA ATP-binding interface. The FeS (orange) and the Arch (purple) domains are inserted into HD1. The boundaries of the FeS cluster binding domain are indicated by red spheres. The human enzyme C-terminal (grey) extension (CTE) is indicated in grey. Domain boundaries are indicated by residue numbers. B,C) 3D representation of the native (cyan) and mutant (pink) overlayed ERCC2 protein structures show a detailed structural environment of the wild-type (green), Arg487 and Asp423 residues in comparison to the Arg487Trp and Asp423Asn mutants (red). Surrounding amino acids (AAs) are indicated as licorice. (B) Note the significant changes in the AA constellations Arg424, Thr425 induced by the by the Asp423Asn replacement. (C) The Arg487Trp AA replacement introduces a tryptophan residue which protrudes beyond the protein surface and might destabilize the interactions with the surrounding AAs His700, Glu690 and Leu701 within the protein loop.
(TIF)

S2 Fig Distribution of PhyloP and CADD scores for 1000G, ClinVar and the mutations identified in this study in the ERCC2 gene. A) Evolutionary conservations (PhyloP) and Combined Annotation Dependent Depletion (CADD) scores are represented for all non-synonymous ERCC2 variants found in BC/OC patients. Blue: Variants with no significant functional effect; Red: variants which showed a deleterious functional effect by no complementation of NER-deficient cells and/or negative modulation of transcription; Green: variants not tested. B) This analysis was further extended to analyze these combined scores for all non-synonymous variants reported in 1000G and ClinVar with no reported clinical significance (Class 1–3), or ClinVar reported pathogenic variants (Class 4–5) to visualize the probability for the ERCC2 variants which have not been functionally tested to be pathogenic or benign. Heat maps show the distribution and frequency for the combined PhyloP and CADD scores in 1000G and ClinVar. Red colors indicate a low frequency and green colors a high frequency. ERCC2 variants showing no functional pathogenic effect (circle), pathogenic variants with NER complementation failure and/or negative modulation of transcription (triangles), and variants not tested in our functional studies (black square) are represented. ERCC2 variants with deleterious functional effects show a better overlap with ClinVar pathogenic variants (Class 4–5) by their location mostly restricted to dark green and yellow as indicated. In contrast, location of variants shows within the dark red plot region when compared to 1000G and ClinVar (Class 1–3).Variants not included in our functional studies show a similar distribution pattern as functional deleterious variants which overlaps with ClinVar pathogenic variants (Class 4–5). In total, most of the ERCC2 variants are located in areas of high conservation and high deleteriousness. Statistical probability scores for these analyses are provided in S4 Table. PhyloP and CADD scores for 1000G and ClinVar variants were obtained from the annotation browser SNiPA [36].
(TIF)

S3 Fig ERCC2 amino acid (AA) sequence alignment. Multiple sequence alignment of protein regions from various species surrounding the identified human ERCC2 missense variants (S4 Table). Affected residues are indicated in red letters. The dotted lines correspond to sequence gaps or sequence regions not yet available. Except Glu167, all affected residues showed strong conservation across vertebrates (Arg166, Gly188, Arg280, Gln316, Asp423, Leu461, Arg487, Val611, Val678, Ala717, Arg722) or even across all species (Pro13, Pro215, Arg450, D513, Val536, Glu576, Arg592, Arg601, Arg631). The AA variability at codon 167 is in line with the results of the effect prediction algorithms which predict the Glu167Gln replacement as benign (S4 Table). Accession number of the ERCC2protein sequences used for AA sequence comparison are as follows: Homo sapiens (NP_000391.1); Pan troglodytes (NP_001233519.1); Macaca mulatta (XP_002808245.1); Canis lupus (XP_541562.3); Bos taurus (NP_001096787.1); Mus musculus (NP_031975.2); Rattus norvegicus (NP_001166280.1); Xenopus tropicalis (NP_001008131.1); Danio rerio (NP_957220.1); Drosophila melanogaster (NP_726036.2); Anopheles gambiae (XP_311900.4); Caenorhabditis elegans (NP_497182.2); Saccharomyces cerevisiae (NP_011098.3); Kluyveromyces lactis (XP_452994.1); Eremothecium gossypii (NP_986780.1); Schizosaccharomyces pombe (NP_593025.1); Magnaporthe oryzae (XP_003716866.1); Neurospora crassa (XP_956536.2); Arabidopsis thaliana (NP_171818.1); and Oryza sativa (NP_001054627.1).
(TIF)

S1 Table Genes covered by the TruSight-Cancer gene panel. (DOCX)

S2 Table ERCC2/XPD primers for Sanger-validation of NGS derived mutations and analysis of familial segregation. All sequences shown in 5’ → 3’ direction. XPD = alternate name of *ERCC2*.
(DOCX)

S3 Table Primer pairs used for PCR amplification, Sanger sequencing, and site directed mutagenesis of ERCC2 variants. All primers (de-salted and deprotected) were synthesized by Sigma-Aldrich (Taufkirchen, Germany).
(DOCX)

S4 Table Effect prediction of ERCC2 missense variants. The probability of effect of non-synonymous mutations in ERCC2 was predicted by the computer programs: SIFT, Sorting Invariant from Tolerated (Score under 0,05: not tolerated; Range 0**–**1); PolyPhen-2, Classification following PSIC scores (HumVar, "benign"**-** "possibly damaging“**—**"probably damaging ", Range: 0**–**1); Provean, Protein Variation Effect Analyze; MAPP, Multivariate Analysis of Protein Polymorphism; Align-GVGD, Scores (C0, C15, C25, C35, C45, C55, C65) from C0 (likely benign) to C65 (likely pathogenic); CADD, Combined Annotation Dependent Depletion [12]. Dlt **=** deleterious, PrD **=** probably damaging, PsD **=** possibly damaging, Bgn **=** benign, Ntr **=** neutral. Conservation was calculated with PhyloP (Score range from -14.1 to 6.4). Grantham [35] distance scores (Range 0**–**215). AA exchanges in gray background are located in cis and form a haplotype.
(DOCX)
