# Supplementary figures and images for: Identification and Functional Testing of ERCC2 Mutations in a Multi-national Cohort of Patients with Familial Breast- and Ovarian Cancer
Source: PLoS Genet. 2016 Aug 9;12(8):e1006248. doi: 10.1371/journal.pgen.1006248 (PMC4978395; doi:10.1371/journal.pgen.1006248)

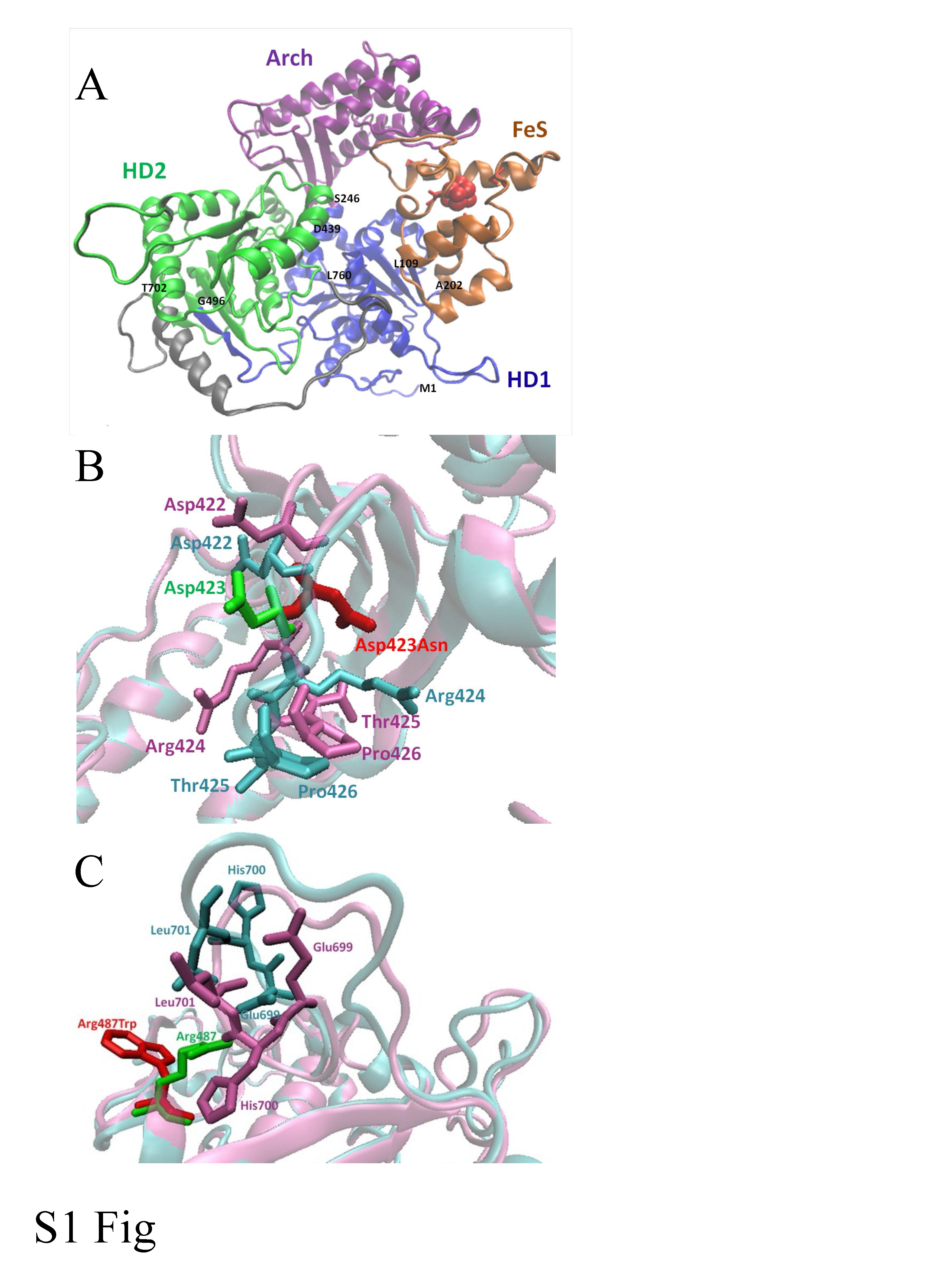

Supplement: S1 Fig — A) Schematic showing the domain structure and canonical motifs of human ERCC2. Helicase motor domains HD1 (blue) and HD2 (green) form the DNA ATP-binding interface. The FeS (orange) and the Arch (purple) domains are inserted into HD1. The boundaries of the FeS cluster binding domain are indicated by red spheres. The human enzyme C-terminal (grey) extension (CTE) is indicated in grey. Domain boundaries are indicated by residue numbers. B,C) 3D representation of the native (cyan) and mutant (pink) overlayed ERCC2 protein structures show a detailed structural environment of the wild-type (green), Arg487 and Asp423 residues in comparison to the Arg487Trp and Asp423Asn mutants (red). Surrounding amino acids (AAs) are indicated as licorice. (B) Note the significant changes in the AA constellations Arg424, Thr425 induced by the by the Asp423Asn replacement. (C) The Arg487Trp AA replacement introduces a tryptophan residue which protrudes beyond the protein surface and might destabilize the interactions with the surrounding AAs His700, Glu690 and Leu701 within the protein loop. (TIF) [file pgen.1006248.s001.tif]

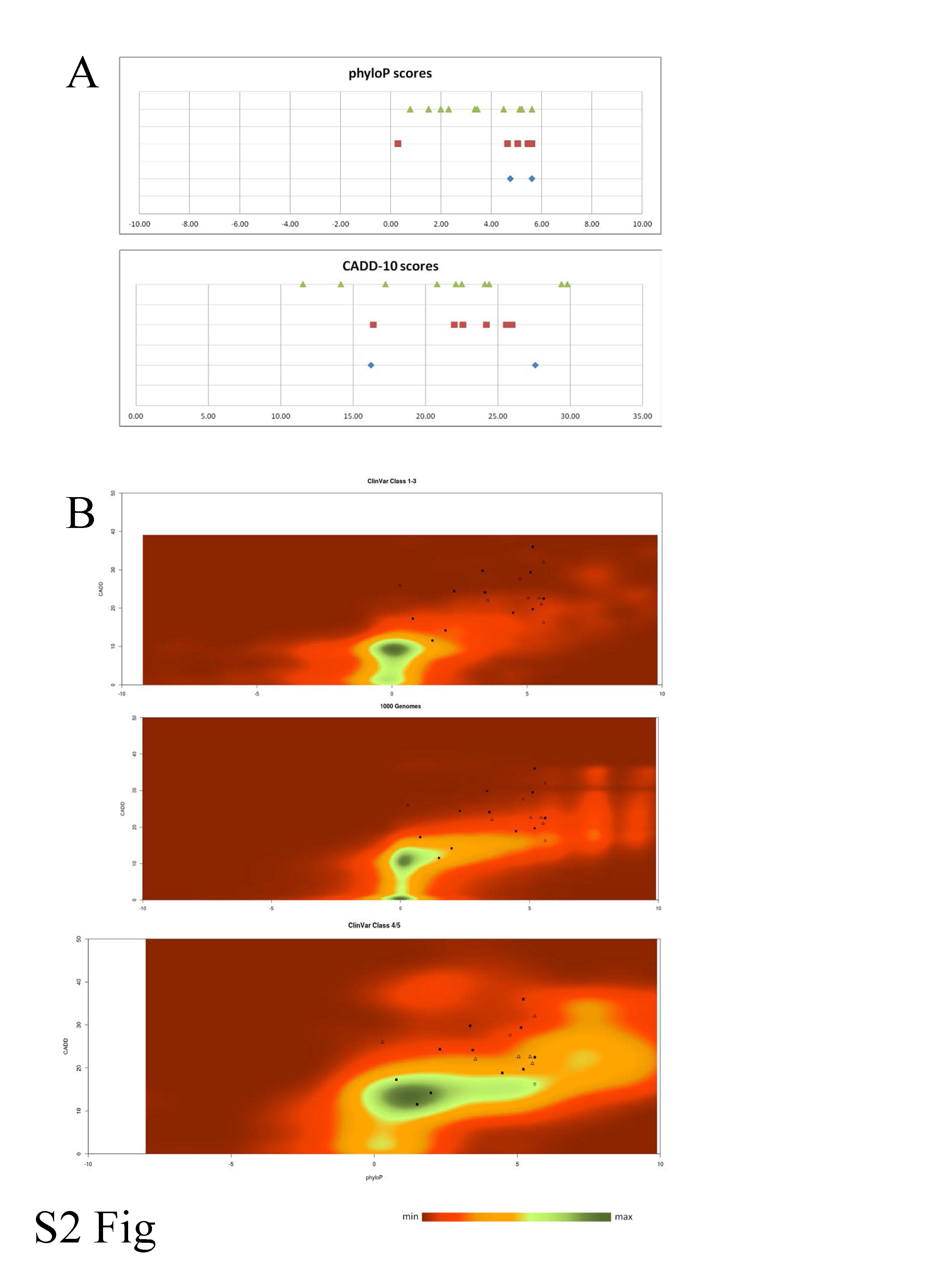

Supplement: S2 Fig — A) Evolutionary conservations (PhyloP) and Combined Annotation Dependent Depletion (CADD) scores are represented for all non-synonymous ERCC2 variants found in BC/OC patients. Blue: Variants with no significant functional effect; Red: variants which showed a deleterious functional effect by no complementation of NER-deficient cells and/or negative modulation of transcription; Green: variants not tested. B) This analysis was further extended to analyze these combined scores for all non-synonymous variants reported in 1000G and ClinVar with no reported clinical significance (Class 1–3), or ClinVar reported pathogenic variants (Class 4–5) to visualize the probability for the ERCC2 variants which have not been functionally tested to be pathogenic or benign. Heat maps show the distribution and frequency for the combined PhyloP and CADD scores in 1000G and ClinVar. Red colors indicate a low frequency and green colors a high frequency. ERCC2 variants showing no functional pathogenic effect (circle), pathogenic variants with NER complementation failure and/or negative modulation of transcription (triangles), and variants not tested in our functional studies (black square) are represented. ERCC2 variants with deleterious functional effects show a better overlap with ClinVar pathogenic variants (Class 4–5) by their location mostly restricted to dark green and yellow as indicated. In contrast, location of variants shows within the dark red plot region when compared to 1000G and ClinVar (Class 1–3).Variants not included in our functional studies show a similar distribution pattern as functional deleterious variants which overlaps with ClinVar pathogenic variants (Class 4–5). In total, most of the ERCC2 variants are located in areas of high conservation and high deleteriousness. Statistical probability scores for these analyses are provided in S4 Table. PhyloP and CADD scores for 1000G and ClinVar variants were obtained from the annotation browser SNiPA [36]. (TIF) [file pgen.1006248.s002.tif]

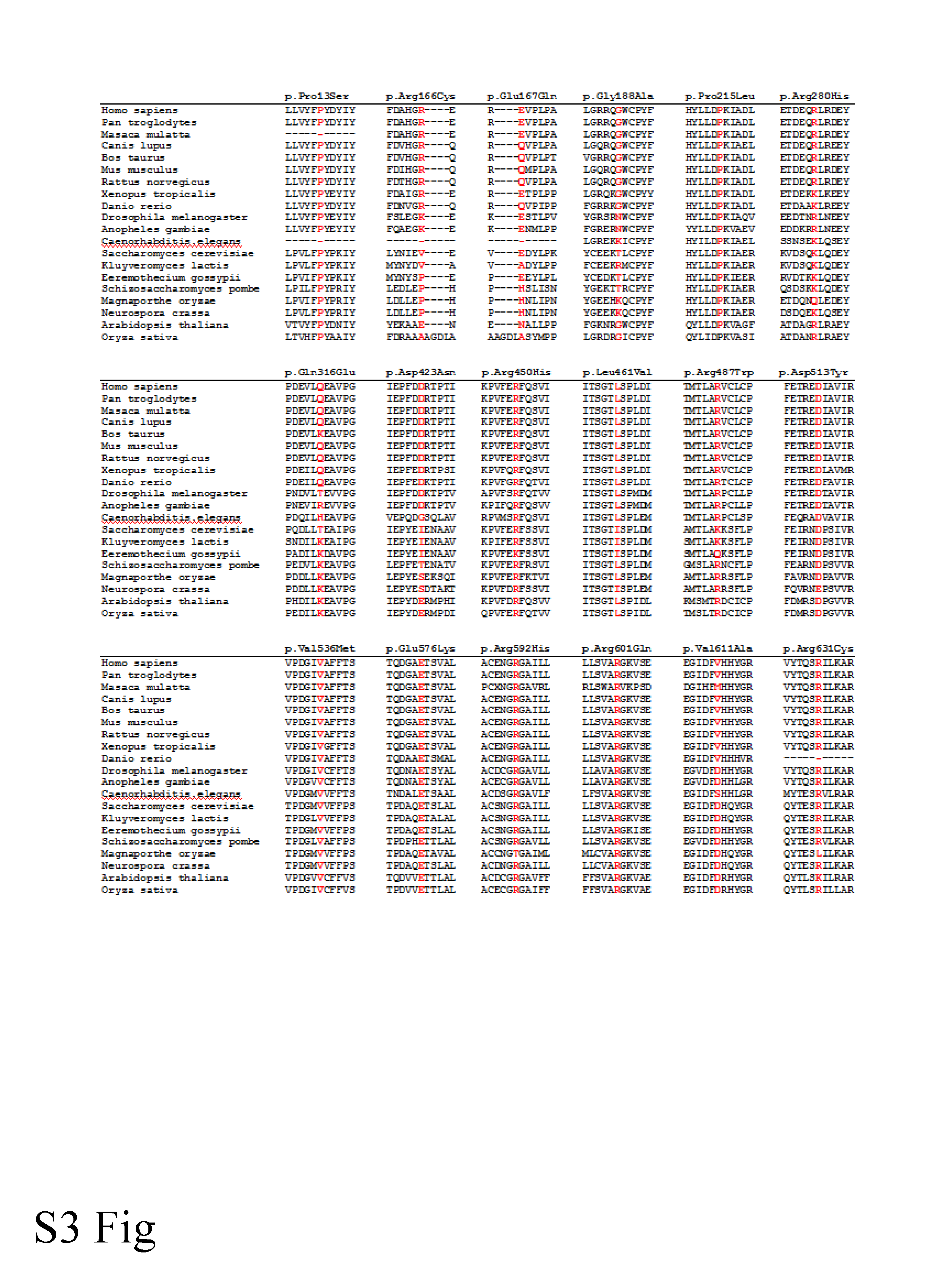

Supplement: S3 Fig — Multiple sequence alignment of protein regions from various species surrounding the identified human ERCC2 missense variants (S4 Table). Affected residues are indicated in red letters. The dotted lines correspond to sequence gaps or sequence regions not yet available. Except Glu167, all affected residues showed strong conservation across vertebrates (Arg166, Gly188, Arg280, Gln316, Asp423, Leu461, Arg487, Val611, Val678, Ala717, Arg722) or even across all species (Pro13, Pro215, Arg450, D513, Val536, Glu576, Arg592, Arg601, Arg631). The AA variability at codon 167 is in line with the results of the effect prediction algorithms which predict the Glu167Gln replacement as benign (S4 Table). Accession number of the ERCC2protein sequences used for AA sequence comparison are as follows: Homo sapiens (NP_000391.1); Pan troglodytes (NP_001233519.1); Macaca mulatta (XP_002808245.1); Canis lupus (XP_541562.3); Bos taurus (NP_001096787.1); Mus musculus (NP_031975.2); Rattus norvegicus (NP_001166280.1); Xenopus tropicalis (NP_001008131.1); Danio rerio (NP_957220.1); Drosophila melanogaster (NP_726036.2); Anopheles gambiae (XP_311900.4); Caenorhabditis elegans (NP_497182.2); Saccharomyces cerevisiae (NP_011098.3); Kluyveromyces lactis (XP_452994.1); Eremothecium gossypii (NP_986780.1); Schizosaccharomyces pombe (NP_593025.1); Magnaporthe oryzae (XP_003716866.1); Neurospora crassa (XP_956536.2); Arabidopsis thaliana (NP_171818.1); and Oryza sativa (NP_001054627.1). (TIF) [file pgen.1006248.s003.tif]
