# Supplementary material for: Identification and Functional Testing of ERCC2 Mutations in a Multi-national Cohort of Patients with Familial Breast- and Ovarian Cancer
Source: PLoS Genet. 2016 Aug 9;12(8):e1006248. doi: 10.1371/journal.pgen.1006248 (PMC4978395; doi:10.1371/journal.pgen.1006248)
Supplement: S1 Table — (DOCX) [file pgen.1006248.s004.docx]

| Gene | RefSeq Transcript | OMIM | Gene | RefSeq Transcript | OMIM |
| --- | --- | --- | --- | --- | --- |
| AIP | NM_003977.2 | [605555](http://www.omim.org/605555) | **HRAS** | NM_005343.2 | [190020](http://www.omim.org/190020) |
| ALK | NM_004304.4 | [105590](http://www.omim.org/105590) | **KIT** | NM_000222.2 | [164920](http://www.omim.org/164920) |
| APC | NM_000038.5 | [611731](http://www.omim.org/611731) | **MAX** | NM_002382.3 | [154950](http://www.omim.org/154950) |
| ATM | NM_000051.3 | [607585](http://www.omim.org/607585) | **MEN1** | NM_130799.2 | [613733](http://www.omim.org/613733) |
| BAP1 | NM_004656.2 | [603089](http://www.omim.org/603089) | **MET** | NM_001127500.1 | [164860](http://www.omim.org/164860) |
| BLM | NM_000057.2 | [604610](http://www.omim.org/604610) | **MLH1** | NM_000249.3 | [120436](http://www.omim.org/120436) |
| BMPR1A | NM_004329.2 | [601299](http://www.omim.org/601299) | **MSH2** | NM_000251.1 | [609309](http://www.omim.org/609309) |
| BRCA1 | NM_007294.3 | [113705](http://www.omim.org/113705) | **MSH6** | NM_000179.2 | [600678](http://www.omim.org/600678) |
| BRCA2 | NM_000059.3 | [600185](http://www.omim.org/600185) | **MUTYH** | NM_001128425.1 | [604933](http://www.omim.org/604933) |
| BRIP1 | NM_032043.2 | [605882](http://www.omim.org/605882) | **NBN** | NM_002485.4 | [602667](http://www.omim.org/602667) |
| BUB1B | NM_001211.5 | [602860](http://www.omim.org/602860) | **NF1** | NM_000267.3 | [613113](http://www.omim.org/613113) |
| CDC73 | NM_024529.4 | [607393](http://www.omim.org/607393) | **NF2** | NM_000268.3 | [607379](http://www.omim.org/607379) |
| CDH1 | NM_004360.3 | [192090](http://www.omim.org/192090) | **NSD1** | NM_022455.4 | [606681](http://www.omim.org/606681) |
| CDK4 | NM_000075.2 | [123829](http://www.omim.org/123829) | **PALB2** | NM_024675.3 | [610355](http://www.omim.org/610355) |
| CDKN1C | NM_000076.2 | [600856](http://www.omim.org/600856) | **PHOX2B** | NM_003924.3 | [603851](http://www.omim.org/603851) |
| CDKN2A | NM_000077.4 | [600160](http://www.omim.org/600160) | **PMS1** | NM_000534.4 | [600258](http://www.omim.org/600258) |
| CEBPA | NM_004364.3 | [116897](http://www.omim.org/116897) | **PMS2** | NM_000535.5 | [600259](http://www.omim.org/600259) |
| CEP57 | NM_014679.3 | [607951](http://www.omim.org/607951) | **PRF1** | NM_001083116.1 | [170280](http://www.omim.org/170280) |
| CHEK2 | NM_007194.3 | [604373](http://www.omim.org/604373) | **PRKAR1A** | NM_002734.3 | [188830](http://www.omim.org/188830) |
| CYLD | NM_015247.2 | [605018](http://www.omim.org/605018) | **PTCH1** | NM_000264.3 | [601309](http://www.omim.org/601309) |
| DDB2 | NM_000107.2 | [600811](http://www.omim.org/600811) | **PTEN** | NM_000314.4 | [601728](http://www.omim.org/601728) |
| DICER1 | NM_177438.2 | [606241](http://www.omim.org/606241) | **RAD51C** | NM_058216.1 | [602774](http://www.omim.org/602774) |
| DIS3L2 | NM_152383.4 | [614184](http://www.omim.org/614184) | **RAD51D** | NM_002878.3 | [602954](http://www.omim.org/602954) |
| EGFR | NM_005228.3 | [131550](http://www.omim.org/131550) | **RB1** | NM_000321.2 | [614041](http://www.omim.org/614041) |
| EPCAM | NM_002354.2 | [185535](http://www.omim.org/185535) | **RECQL4** | NM_004260.3 | [603780](http://www.omim.org/603780) |
| ERCC2 | NM_000400.3 | [126340](http://www.omim.org/126340) | **RET** | NM_020975.4 | [164761](http://www.omim.org/164761) |
| ERCC3 | NM_000122.1 | [133510](http://www.omim.org/133510) | **RHBDF2** | NM_024599.5 | [614404](http://www.omim.org/614404) |
| ERCC4 | NM_005236.2 | [133520](http://www.omim.org/133520) | **RUNX1** | NM_001754.4 | [151385](http://www.omim.org/151385) |
| ERCC5 | NM_000123.3 | [133530](http://www.omim.org/133530) | **SBDS** | NM_016038.2 | [607444](http://www.omim.org/607444) |
| EXT1 | NM_000127.2 | [608177](http://www.omim.org/608177) | **SDHAF2** | NM_017841.2 | [613019](http://www.omim.org/613019) |
| EXT2 | NM_207122.1 | [608210](http://www.omim.org/608210) | **SDHB** | NM_003000.2 | [185470](http://www.omim.org/185470) |
| EZH2 | NM_004456.4 | [601573](http://www.omim.org/601573) | **SDHC** | NM_003001.3 | [602413](http://www.omim.org/602413) |
| FANCA | NM_000135.2 | [607139](http://www.omim.org/607139) | **SDHD** | NM_003002.2 | [602690](http://www.omim.org/602690) |
| FANCB | NM_001018113.1 | [300515](http://www.omim.org/300515) | **SLX4** | NM_032444.2 | [613278](http://www.omim.org/613278) |
| FANCC | NM_000136.2 | [613899](http://www.omim.org/613899) | **SMAD4** | NM_005359.5 | [600993](http://www.omim.org/600993) |
| FANCD2 | NM_033084.3 | [613984](http://www.omim.org/613984) | **SMARCB1** | NM_003073.3 | [601607](http://www.omim.org/601607) |
| FANCE | NM_021922.2 | [613976](http://www.omim.org/613976) | **STK11** | NM_000455.4 | [602216](http://www.omim.org/602216) |
| FANCF | NM_022725.3 | [613897](http://www.omim.org/613897) | **SUFU** | NM_016169.3 | [607035](http://www.omim.org/607035) |
| FANCG | NM_004629.1 | [602956](http://www.omim.org/602956) | **TMEM127** | NM_017849.3 | [613403](http://www.omim.org/613403) |
| FANCI | NM_001113378.1 | [611360](http://www.omim.org/611360) | **TP53** | NM_000546.4 | [191170](http://www.omim.org/191170) |
| FANCL | NM_018062.3 | [608111](http://www.omim.org/608111) | **TSC1** | NM_000368.4 | [605284](http://www.omim.org/605284) |
| FANCM | NM_020937.2 | [609644](http://www.omim.org/609644) | **TSC2** | NM_000548.3 | [191092](http://www.omim.org/191092) |
| FH | NM_000143.3 | [136850](http://www.omim.org/136850) | **VHL** | NM_000551.3 | [608537](http://www.omim.org/608537) |
| FLCN | NM_144997.5 | [607273](http://www.omim.org/607273) | **WRN** | NM_000553.4 | [604611](http://www.omim.org/604611) |
| GATA2 | NM_032638.4 | [137295](http://www.omim.org/137295) | **WT1** | NM_024426.4 | [607102](http://www.omim.org/607102) |
| GPC3 | NM_004484.3 | [300037](http://www.omim.org/300037) | **XPA** | NM_000380.3 | [611153](http://www.omim.org/611153) |
| HNF1A | NM_000545.5 | [142410](http://www.omim.org/142410) | **XPC** | NM_004628.4 | [613208](http://www.omim.org/613208) |
