# Supplementary material for: Identification and Functional Testing of ERCC2 Mutations in a Multi-national Cohort of Patients with Familial Breast- and Ovarian Cancer
Source: PLoS Genet. 2016 Aug 9;12(8):e1006248. doi: 10.1371/journal.pgen.1006248 (PMC4978395; doi:10.1371/journal.pgen.1006248)
Supplement: S2 Table — All sequences shown in 5’ → 3’ direction. XPD = alternate name of ERCC2. (DOCX) [file pgen.1006248.s005.docx]

| Name | SEQUENCE | hg19 coordinates |
| --- | --- | --- |
| XPD_ex1_2_F | AATATAGGTGGAGCGAGCCC | chr19:45,873,881-45,873,900 |
| XPD_ex1_2_R | GGAAGATTATGGGGTCCTGG | chr19:45,873,260-45,873,279 |
| XPD_ex3_4_F | CTGCTGCTGTGTTATCCTGC | chr19:45,872,508-45,872,527 |
| XPD_ex3_4_R | CAGTCTCTTCCCCAGCTTCC | chr19:45,872,072-45,872,091 |
| XPD_ex5_F | TAAGCTGGGACTCATCCTGG | chr19:45,872,167-45,872,186 |
| XPD_ex5_R | GACTCCCTGGAGGAAGTGTG | chr19:45,871,699-45,871,718 |
| XPD_ex6_7_F | CAAGTTGTCCAAAACCCCAG | chr19:45,868,501-45,868,520 |
| XPD_ex6_7_R | ACCAACAGGGAGATGCAGAC | chr19:45,867,971-45,867,990 |
| XPD_ex8_9_F | ATACTTCTGCCTGGCCTGTG | chr19:45,867,911-45,867,930 |
| XPD_ex8_9_R | CTCTTTGATCCTGCGGAGAG | chr19:45,867,368-45,867,387 |
| XPD_ex10_11_F | CTGCAGAAGACGGTGCTCAG | chr19:45,867,493-45,867,512 |
| XPD_ex10_11_R | TCACTCACTCCTGATGCTGC | chr19:45,866,889-45,866,908 |
| XPD_ex12_F | TGGCTATTTGTGACATGCAG | chr19:45,865,069-45,865,088 |
| XPD_ex12_R | AAAATAGGGCCCACACTTCC | chr19:45,864,650-45,864,669 |
| XPD_ex13_14_15_F | CAGTACGGGGTGGAGATTGG | chr19:45,861,051-45,861,070 |
| XPD_ex13_14_15_R | CCTCTTCGCTGTAAAGCTCTC | chr19:45,860,417-45,860,437 |
| XPD_ex16_F | TGCAGATAACAGGGTTGCTG | chr19:45,859,256-45,859,275 |
| XPD_ex16_R | TATGCTGCCTCTGACACACC | chr19:45,858,703-45,858,722 |
| XPD_ex17_F | GGGGATATGTGTGAAAAGCC | chr19:45,858,212-45,858,231 |
| XPD_ex17_R | CACACAGGAAACCTGTCACC | chr19:45,857,879-45,857,898 |
| XPD_ex18_19_F | CAGAGACATGGTGATGTGGG | chr19:45,856,669-45,856,688 |
| XPD_ex18_19_R | GTTACAAGTGTGGCTGGTGG | chr19:45,856,105-45,856,124 |
| XPD_ex20_21_F | GAGCTCTCTGGAACACCTGC | chr19:45,856,282-45,856,301 |
| XPD_ex20_21_R | CAGGGACAGAAGGTCATTCG | chr19:45,855,631-45,855,650 |
| XPD_ex22_F | CTGTTTCCCGTTCATTTCCC | chr19:45,855,692-45,855,711 |
| XPD_ex22_R | CTCTTTGCTGAGGGCAGGAG | chr19:45,855,347-45,855,366 |
| XPD_ex23_F | CCGCTCTGGATTATACGGAC | chr19:45,855,105-45,855,124 |
| XPD_ex23_R | CATAAGACCTTCTAGCACCACC | chr19:45,854,795-45,854,816 |
