# Supplementary material for: Identification and Functional Testing of ERCC2 Mutations in a Multi-national Cohort of Patients with Familial Breast- and Ovarian Cancer
Source: PLoS Genet. 2016 Aug 9;12(8):e1006248. doi: 10.1371/journal.pgen.1006248 (PMC4978395; doi:10.1371/journal.pgen.1006248)
Supplement: S3 Table — All primers (de-salted and deprotected) were synthesized by Sigma-Aldrich (Taufkirchen, Germany). (DOCX) [file pgen.1006248.s006.docx]

| **Application** | **Name of primer** | **Sequence (5‘ -> 3‘)** |
| --- | --- | --- |
| Cloning of wt-*ERCC2* | XPD-For2-KpnI | TTAGGTACCATGAAGCTCAACGTGGACG |
|  | XPD-Rev2-XbaI | TTATCTAGATCAGAGCTGCTGAGCAATCT |
| Sequencing | T7 | TAATACGACTCACTATAGGG |
|  | SeqD21 | GCCAATGTGGTGGTTTATAGCT |
|  | seqSS | CCCCTCAGATTCTGTGCT |
|  | SeqD31 | ATCGAGCCCTTTGACG |
|  | SeqD41 | CAAAGTGTCCGAGGGAATCG |
|  | SeqD32r | ACCAGTTCCAGATTCGTGAGA |
|  | M13 | TAGAAGGCACAGTCGAGG |
| Mutagenesis | R601W | ForCCTGCTGTCAGTGGCCTGGGGC |
|  |  | RevCCCTCGGACACTTTGCCCCAGGC |
|  | F568YFS | ForCCAGAGGAACAAGCTGCTCTATTGAGACC |
|  |  | RevGGCACCATCCTGGGTCTCAATAGAGC |
|  | Ser746FS | ForGGAGCAGCTAGAATCGAGGAGACGCTGAAG |
|  |  | RevCTTCAGCGTCTCCTCGATTCTAGCTGCTCC |
|  | Asp513Tyr | ForTTTGAGACCCGGGAGTATATTGCTGTGATCC |
|  |  | RevGGATCACAGCAATATACTCCCGGGTCTCAAA |
|  | R631C | ForCGTCTACACACAGAGCTGCATTC |
|  |  | RevCGCGCCTTGAGAATGCAGCTCTG |
|  | Arg487Trp | ForCATGACGCTGGCATGGGTCT |
|  |  | RevAGAGGCAGACCCATGCCA |
|  | Val536Met | ForCCGCTGTGGTCCCTGATGGCATCATGGCC |
|  |  | RevGGTAGCTGGTGAAGAAGGCCATGATGCC |
|  | Arg601Gln | ForCCTGCTGTCAGTGGCCCAGGGC |
|  |  | RevCCCTCGGACACTTTGCCCTGGGC |
|  | R592H | ForCAGGAGGCCTGCGAGAATGGCCACGGG |
|  |  | RevCTGACAGCAGGATGGCCCCGTGGC |
|  | Arg450His | ForCCATCAAACCCGTATTTGAGCATTTCC |
|  |  | RevGTGATGATGACAGACTGGAAATGCTCA |
|  | L461V | ForGTCATCATCACATCTGGGACAGTGTCCC |
|  |  | RevGGGTAGATGTCCAGCGGGGACACTGTCCC |
|  | A717G | ForGGACGAGGGTGTCCAGGTGGGCAAG |
|  |  | RevCATCTGCCGCAGGAAGTACTTGCCCACC |
|  | D423N | ForCATCGAGCCCTTTGACAACAG |
|  |  | RevGGTCGGGGTTCTGTTGTCAAAG |
