# Supplementary material for: Identification and Functional Testing of ERCC2 Mutations in a Multi-national Cohort of Patients with Familial Breast- and Ovarian Cancer
Source: PLoS Genet. 2016 Aug 9;12(8):e1006248. doi: 10.1371/journal.pgen.1006248 (PMC4978395; doi:10.1371/journal.pgen.1006248)
Supplement: S4 Table — The probability of effect of non-synonymous mutations in ERCC2 was predicted by the computer programs: SIFT, Sorting Invariant from Tolerated (Score under 0,05: not tolerated; Range 0–1); PolyPhen-2, Classification following PSIC scores (HumVar, "benign"- "possibly damaging“—"probably damaging ", Range: 0–1); Provean, Protein Variation Effect Analyze; MAPP, Multivariate Analysis of Protein Polymorphism; Align-GVGD, Scores (C0, C15, C25, C35, C45, C55, C65) from C0 (likely benign) to C65 (likely pathogenic); CADD, Combined Annotation Dependent Depletion [12]. Dlt = deleterious, PrD = probably damaging, PsD = possibly damaging, Bgn = benign, Ntr = neutral. Conservation was calculated with PhyloP (Score range from -14.1 to 6.4). Grantham [35] distance scores (Range 0–215). AA exchanges in gray background are located in cis and form a haplotype. (DOCX) [file pgen.1006248.s007.docx]

| AA change  (N = 20) | SIFT | PPH2 | Pro- vean | MAPP | Align GVGD | CADD | PhyloP | Grantham  Distance | Summary |
| --- | --- | --- | --- | --- | --- | --- | --- | --- | --- |
| Pro13Ser | Dlt | PrD | Dlt | bad | C0 | 19.70 | 5.21 | 74 | pathogenic |
| Arg166Cys | Dlt | PrD | Dlt | bad | C0 | 29.80 | 3.35 | 180 | pathogenic |
| Glu167Gln | Tol | Bgn | Ntr | good | C0 | 11.52 | 1.5 | 29 | benign |
| Gly188Ala | Tol | Bgn | Ntr | good | C0 | 24.10 | 3.44 | 60 | benign |
| Pro215Leu | Dlt | PrD | Dlt | bad | C65 | 29.40 | 5.13 | 98 | pathogenic |
| Arg280His | Dlt | Bgn | Dlt | bad | C0 | 24.40 | 2.30 | 29 | pathogenic |
| Gln316Glu | Tol | Bgn | Ntr | good | C0 | 14.16 | 1.98 | 29 | benign |
| Asp423Asn | Tol | Bgn | Ntr | good | C0 | 21.00 | 5.53 | 23 | benign |
| Arg450His | Dlt | Bgn | Dlt | bad | C0 | 27.60 | 4.75 | 29 | pathogenic |
| Leu461Val | Dlt | PsD | Ntr | good | C0 | 25.60 | 4.64 | 32 | benign |
| Arg487Trp | Dlt | PrD | Dlt | bad | C0 | 26.00 | 0.29 | 101 | pathogenic |
| Asp513Tyr | Dlt | PrD | Dlt | good | C25 | 22.60 | 5.05 | 160 | pathogenic |
| Val536Met | Dlt | PrD | Dlt | bad | C15 | 22.60 | 5.45 | 21 | pathogenic |
| Glu576Lys | Dlt | PrD | Dlt | bad | C55 | 22.50 | 5.61 | 56 | pathogenic |
| Arg592His | Dlt | PrD | Dlt | bad | C25 | 16.23 | 5.61 | 29 | pathogenic |
| Arg601Gln | Dlt | PsD | Dlt | bad | C35 | 32.00 | 5.61 | 43 | pathogenic |
| Val611Ala | Tol | Bgn | Ntr | good | C0 | 18.83 | 4.48 | 64 | benign |
| Arg631Cys | Dlt | PrD | Dlt | bad | C65 | 22.00 | 3.54 | 180 | pathogenic |
| Val678Leu | Tol | Bgn | Ntr | good | C0 | 17.23 | 0.77 | 32 | benign |
| Arg722Gln | Dlt | PsD | Dlt | bad | C0 | 36.00 | 5.21 | 43 | pathogenic |
